# Reciprocal relationship between APP positioning relative to the membrane and PS1 conformation

**DOI:** 10.1186/1750-1326-6-15

**Published:** 2011-02-10

**Authors:** Kengo Uemura, Katherine C Farner, Navine Nasser-Ghodsi, Phill Jones, Oksana Berezovska

**Affiliations:** 1 Alzheimer Research Unit, MassGeneral Institute for Neurodegenerative Diseases, Massachusetts General Hospital, Charlestown, MA 02129, USA; 2 Kyoto University, Graduate School of Medicine, Japan

## Abstract

**Background:**

Several familial Alzheimer disease (FAD) mutations within the transmembrane region of the amyloid precursor protein (APP) increase the Aβ_42/40 _ratio without increasing total Aβ production. In the present study, we analyzed the impact of FAD mutations and γ-secretase modulators (GSMs) that alter the Aβ_42/40 _ratio on APP C-terminus (CT) positioning relative to the membrane, reasoning that changes in the alignment of the APP intramembranous domain and presenilin 1 (PS1) may impact the PS1/γ-secretase cleavage site on APP.

**Results:**

By using a Förster resonance energy transfer (FRET)-based technique, fluorescent lifetime imaging microscopy (FLIM), we show that Aβ_42/40 _ratio-modulating factors which target either APP substrate or PS1/γ-secretase affect proximity of the APP-CT to the membrane and change PS1 conformation.

**Conclusions:**

Thus, we propose that there is a reciprocal relationship between APP-CT positioning relative to the membrane and PS1 conformation, suggesting that factors that modulate either APP positioning in the membrane or PS1 conformation could be exploited therapeutically.

## Background

γ-Secretase is responsible for cleavage of a number of type I membrane proteins, including amyloid precursor protein (APP) and Notch, and is comprised of presenilin 1 or 2, Aph1, Pen2 and Nicastrin [1-5]. Proteolytic processing of APP by β- and γ-secretases results in production of amyloid β (Aβ) peptides. The major Aβ species are 40 and 42 amino acid long peptides, the latter of which is recognized as the more toxic species involved in Alzheimer's disease (AD) pathogenesis [2,6,7]. Factors that modulate the Aβ_42/40 _ratio can be classified into at least two categories; 1) substrate-targeting manipulations, such as FAD-linked mutations within the intramembranous region of the APP substrate [8,9], and 2) γ-secretase-targeting modifications, such as FAD-linked PS1 mutations [10-12], Pen-2 N-terminus modification [13] or expression of different Aph1 isoform [14]. In addition, treatment with pharmacological agents, γ-secretase modulators (GSMs), could alter the Aβ_42/40 _ratio [15-17]. However, there is a controversy whether the primary target of these compounds is APP substrate [18-20], or PS1/γ-secretase [21-25].

Using Förster resonance energy transfer (FRET)/fluorescent lifetime imaging microscopy (FLIM) technique, we have previously demonstrated that PS1, a catalytic site of γ-secretase, could exist in a "closed" (close proximity between the PS1 N-terminus, C-terminus, and a large cytoplasmic loop domain) and "open" (longer distance between them) conformations [26-28]. Although the detailed molecular mechanism responsible for different PS1 conformational states, and underlying the precision of APP cleavage by PS1/γ-secretase is currently unknown, we found that the "closed" conformation of PS1 is consistently linked to a higher Aβ_42/40 _ratio, whereas the "open" conformation is associated with a lower Aβ_42/40 _ratio [26-28].

Notably, in addition to manipulations directly targeting components of the γ-secretase complex, mutations within the transmembrane region of the APP substrate have been shown to induce changes in the PS1 conformation. For example, APP with FAD-linked V717I or I716F mutations that increase the Aβ_42/40 _ratio seem to associate with the PS1/γ-secretase earlier in the secretory pathway, alter the alignment of APP with PS1, and shift PS1 into a "close" NT and CT proximity conformation [29]. Conversely, the APP V715F substitution, which dramatically decreases the Aβ_40 _and Aβ_42 _while increasing Aβ_38 _levels, induced a structural rearrangements in PS1 reminiscent of that observed after the treatment with Aβ_42 _-lowering non-steroidal anti-inflammatory drugs ("open" conformation) [30].

Based on these findings, we hypothesized that APP-targeting manipulations may alter conformation of the APP molecule or it's positioning within the plane of the membrane. This alteration may change APP substrate presentation to the PS1/γ-secretase, and consequently induce a shift in PS1 conformation. Since previous structural analysis predicts that the APP cytoplasmic domain can associate with the membrane and alter its positioning in response to various stimuli [31], in the current study we analyzed proximity between the membrane and APP-CT as readout of APP transmembrane positioning. Thus, to better understand the relationship between APP CT transmembrane positioning, PS1/γ-secretase conformation, and the Aβ_42/40 _ratio, we asked whether APP-CT proximity to the membrane correlates with the Aβ_42/40 _ratio and can be affected by the PS1 conformational change. The FLIM assay was utilized to monitor relative distance between the two fluorophores labelling membrane and APP-CT in intact cells. We found that FAD mutations within the APP transmembrane domain, that raise the Aβ_42/40 _ratio, increase proximity of the APP-CT to the membrane. Interestingly, treatment of cells with GSMs, which are known to modify the Aβ_42/40 _ratio and induce PS1 conformational change [24,26,28], led to altered APP-CT and membrane proximity only in the presence of PS1/γ-secretase. Surprisingly, we found that Aβ_42/40 _ratio-raising FAD-linked mutations in PS1 also affect the positioning of APP relative to the membrane in a manner similar to that of the FAD-linked APP mutations. These results suggest a reciprocal relationship between conformation of the APP-CT and/or its orientation relative to the membrane and PS1 conformation. Thus, factors that modulate either APP positioning or PS1 conformation could be exploited therapeutically to correct pathogenic Aβ_42/40 _ratio, and thus prevent or slow down progression of AD.

## Results

### The Aβ_42/40 _ratio-raising APP mutations increase the proximity between APP-CT and the membrane

**Figure 1:**
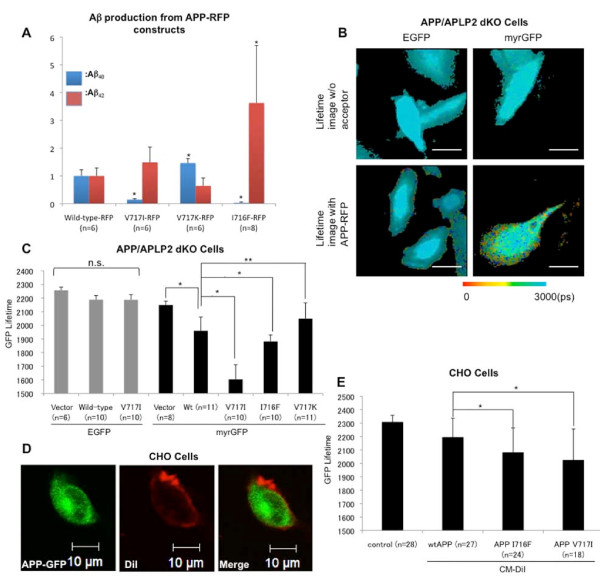
**The Ab42/40 ratio-modulating APP mutations induce changes in APP positioning relative to the membrane **. A) ELISA detection of the human Aβ40 and Aβ42 in conditioned media of the cells transiently transfected with human APP-RFP constructs with designated mutations. The amount of each Aβ species was normalized to that obtained from the cells expressing wild-type APP-RFP (Aβ_40 _≈70 pMol/L, Aβ_42 _≈1 pMol/L). Three independent experiments were performed. (mean ± SD; *p < 0.001 vs. wild-type APP-RFP, ANOVA, n: number of wells in the representative experiment shown). B) FLIM analysis of the proximity between APP-CT RFP and myrGFP labeled membrane. The pseudo-colour images show distribution of the EGFP and myrGFP donor fluorophore lifetimes in the presence (bottom) or absence (top) of the RFP acceptor fluorophore fused to the wild-type APP-CT. Only cells transfected with myrGFP as a donor fluorophore showed lifetime shortening (red and yellow pixels) in the presence of APP-RFP, with the shortest lifetime at the cell periphery. Scale bar: 10 μm. Colorimetric scale shows GFP fluorophore lifetime in picoseconds. C) Quantitative FLIM analysis of the GFP lifetimes in APP/APLP2 dKO cells expressing wild-type and mutant APP-RFP constructs. In cells transfected with the EGFP as a donor fluorophore (grey bars), the donor lifetime did not change significantly in the presence of either wild-type or V717I APP-RFP. In cells transfected with myrGFP as a donor (black bars), the donor lifetime was significantly shortened in the presence of RFP acceptor at the APP CT. FAD-linked APP mutations (V717I and I716F) significantly shortened, whereas V717K APP mutation significantly increased the lifetime of myrGFP donor, compared to that of the wild-type APP (mean ± SD; *p < 0.001, **p < 0.01, ANOVA). Data from one of the three independent experiments is shown; n = cell number. D) CHO cells were transfected with APP-GFP to serve as a donor fluorophore (green) in the FLIM assay. Plasma membrane was stained with CM-DiI to serve as an acceptor fluorophore (red). Merged image shows that APP signal is outlined by red membrane. Scale bar; 10 μm. E) The graph shows average lifetime of GFP donor fluorophore in CHO cells from (D). The lifetime of GFP donor was shortened in cells with CM-DiI membrane staining. In the presence of I716F and V717I mutations, the GFP lifetime was significantly shorter than that in wild-type APP-GFP transfected cells (mean ± SD; *p < 0.01, ANOVA). Three independent experiments were performed (n: total cell number).

First, we measured the effect of V717I, V717K or I716F mutations located in the transmembrane domain of APP on the Aβ_42/40 _ratio in CHO cells transiently transfected with the mutant APP constructs. As reported previously [8,9], the amount of Aβ_42 _was increased while the amount of the Aβ_40 _was decreased in the conditioned medium of V717I and I716F APP expressing cells, leading to a significantly elevated Aβ_42/40 _ratio (**Figure **1A). By contrast, artificial V717K APP mutation [32] significantly lowered the Aβ_42/40 _ratio.

To determine whether these APP mutations affect APP-CT proximity to the membrane, APP-CT-RFP (wild-type or mutant) was co-transfected with either myrGFP or EGFP into APP/APLP2 dKO cells, and the lifetime of GFP donor was measured by FLIM. MyrGFP has a myristoylation sequence that restricts expression of GFP to the membrane [33]. In cells transfected with the myrGFP and wild-type APP-RFP, the lifetime of donor GFP was shortened, compared to that in the myrGFP only expressing cells. This indicates energy transfer from myrGFP to RFP, and close proximity between the APP-CT and membrane. However, in cells transfected with the EGFP without myristoylation signal, the donor lifetime did not change significantly by co-expression with the APP-RFP (**Figure **1B, C), confirming specificity of the FRET signal in the former, and close proximity of the APP CT to the membrane.

Interestingly, cells transfected with APP-RFP mutants that yield high Aβ_42/40 _ratio (V717I and I716F) showed shorter myrGFP donor lifetime, compared to that in cells transfected with the wild-type APP-RFP (**Figure **1C), indicating increased proximity between mutant APP-CT and the membrane. As expected, APP-RFP V717I mutant did not shorten the lifetime of EGFP without myristoylation signal, confirming that the observed shortening of the myrGFP lifetime was specifically caused by the closer proximity of the RFP acceptor at V717I and I716F APP C-termini to the membrane. Conversely, myrGFP donor lifetime was significantly longer in cells expressing Aβ_42/40 _ratio-lowering V717K APP-RFP mutant, compared to that in the wild-type APP-RFP cells (**Figure **1C). The level of expression for different APP mutants is shown in Additional file 1, Figure S1. Please note, APP I716F and V717K mutants are expressed at comparable levels, however, these mutations affect APP-RFP proximity to the membrane in an opposite fashion, as detected by the FLIM assay. Moreover, additional tests were performed to demonstrate that the levels of APP-RFP (Additional file 1, Figure S2) or PS1 (Additional file 1, Figure S3) expression do not affect the myrGFP donor fluorophore lifetime in the FLIM assay. This further confirms that it is the Aβ_42/40 _ratio altering mutations, and not the level of expression, that affect APP CT proximity relative to the membrane.

We used a two-exponential model of the lifetime analysis for the measurement of myrGFP and APP-CT proximity (see Methods and [24,27]). First, the baseline myrGFP lifetime (t1, no FRET) is established in the absence of an acceptor fluorophore. When myrGFP (membrane) and APP-RFP proximity is analysed, the population with a longer lifetime (no FRET) is "fixed", i.e. excluded from the lifetime analysis, and a shorter lifetime of a second, FRET-ing population (t2) is recorded. Thus, parts of the myrGFP labelled membrane that do not contain RFP-labeled APP (or most of the APP molecules not interacting with PS1/γ-secretase, see bellow) have been excluded from the analysis and comparisons between different experimental conditions.

To verify the above finding, we used an alternative strategy by reversing the donor and acceptor fluorophores. GFP fused to the APP-CT served as a donor fluorophore, and CM-DiI was used to stain the membrane, and served as a FRET acceptor (**Figure **1D). As expected, in the presence of CM-DiI staining, the lifetime of GFP was significantly shortened, indicating energy transfer from the GFP to DiI, and close proximity of the APP-CT to the membrane (**Figure **1E). Presence of the Aβ_42 _-raising APP mutations led to a further shortening of the APP-GFP lifetime (**Figure **1E), confirming that APP mutations caused a shift in the APP-CT orientation relative to the membrane.

### γ-Secretase modulators (GSMs) cause a shift in the APP positioning within the membrane

**Figure 2:**
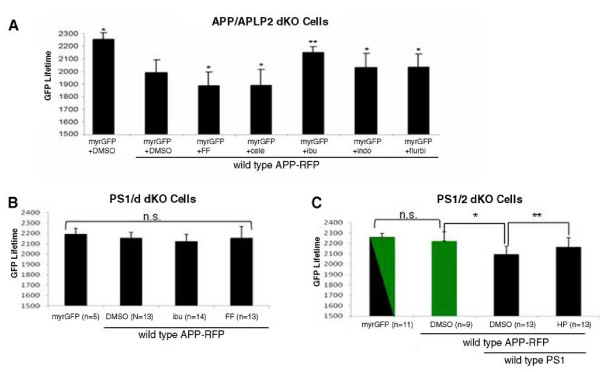
**FLIM analysis of the effect of GSMs and HP on APP-CT positioning relative to the membrane **. A) APP/APLP2 dKO cells co-transfected with myrGFP and wild-type APP-RFP were treated with vehicle control, fenofibrate (FF), celecoxib (cele), ibuprofen (ibu), indomethacin (indo), or flurbiprofen (flurbi). The donor lifetime shortened in cells treated with FF and cele, and increased in cells treated with ibu, indo and flurbi, reflecting increased and decreased proximity between the APP-CT and membrane, respectively, compared to that in cells treated with the DMSO vehicle. mean ± SD; *p < 0.05; **p < 0.001, ANOVA; n = 42-84 cells analyzed per condition in three independent experiments. B) PS1/2 dKO cells were co-transfected with myrGFP and APP-RFP, and treated with fenofibrate or ibuprofen. Neither ibuprofen nor fenofibrate treatment significantly changed the myrGFP lifetime in PS1/2 dKO cells. C) PS1/2 dKO cells (green bar) and PS1/2 dKO + wild-type PS1 cells (black bars) co-transfected with myrGFP and APP-RFP were treated with either DMSO or HP to prevent APP-PS1 interactions. Three to five independent experiments were performed. Data from a representative experiment is shown. n: number of cells analyzed in the experiment (*p < 0.01; **p < 0.05; n.s. - not significant; ANOVA).

It has been reported recently [18,19] that Aβ_42/40 _ratio-modulating GSMs could directly bind to the APP substrate. Thus, we tested whether GSMs affect the Aβ_42/40 _ratio by altering APP membrane positioning. For this, APP/APLP2 dKO cells co-transfected with myrGFP and wild-type APP-RFP were treated with either Aβ_42_-raising (fenofibrate, celecoxib) or Aβ_42_-lowering (ibuprofen, indomethacin, flurbiprofen) GSMs. We found that fenofibrate and celecoxib treatment significantly decreased the lifetime of myrGFP donor, compared to the vehicle control treatment, indicating that they changed positioning of the APP-CT relative to the membrane in the same direction as Aβ_42/40 _ratio-raising APP FAD mutations (**Figure **2A). Conversely, treatment with Aβ_42_-lowering GSMs increased the donor lifetime (**Figure **2A). These findings support the idea that change in the positioning of APP-CT to the membrane reflects a change in the Aβ_42/40 _ratio, with shorter distance between the APP-CT and membrane correlating with a higher, and longer distance with a lower Aβ_42/40 _ratio.

Our previous studies showed that fenofibrate and ibuprofen could also allosterically modify PS1 conformation. To demonstrate whether there is any interplay between the APP-CT position and PS1 conformation, we asked whether GSMs could affect APP-CT orientation in the absence of PS1/γ-secretase. To answer this question, PS1/2 dKO cells co-transfected with myrGFP and wild-type APP-RFP were treated with either ibuprofen or fenofibrate. We found that treatment with neither one of the GSMs affect the lifetime of myrGFP donor in the absence of PS1/2 (**Figure **2B). Moreover, our data indicate that APP-CT locates far from the membrane so that the distance cannot support the energy transfer from myrGFP to RFP on the APP-CT in the absence of presenilins.

To establish whether PS1/γ-secretase presence and/or interaction with the APP substrate are needed to change positioning of APP-CT relative to the membrane, we used the previously characterized helical peptide (HP) γ-secretase inhibitor, which docks to the substrate-binding site on γ-secretase and prevents binding of APP to the PS1/γ-secretase [34]. Wild-type APP-RFP was transfected into either PS1/2 dKO cells or PS1/2 dKO cells reconstituted by stable expression of the wild-type PS1 (PS1/2 dKO + wild-type PS1 cells). Once again, the expression of APP-RFP did not alter the lifetime of donor myrGFP in PS1/2 dKO cells, indicating that APP-CT and the membrane are not close enough to support FRET in the absence of presenilins. On the contrary, the donor lifetime was significantly shortened in PS1/2 dKO + wild-type PS1 cells, indicating that APP-CT proximity to the membrane changes in the presence of PS1. When APP docking to PS1/γ-secretase was inhibited by HP treatment in PS1/2 dKO + wild-type PS1 cells, we observed a significant increase in the myrGFP donor lifetime, confirming that APP-PS1 interaction affects the positioning of APP-CT (**Figure **2C). As mentioned above, the two-exponential FLIM analysis of APP-PS1 interactions, which monitors only those APP molecules that do interact with PS1/γ-secretase, leads to increased sensitivity of the assay to "presenilin effects" on APP-CT orientation towards the membrane.

Similar results were obtained after HP treatment of the APP/APLP2 dKO cells transfected with APP-RFP and expressing wild-type PS1 on endogenous levels (Additional file 1, Figure S4), indicating that APP binding/interaction with the PS1/γ-secretase rearranges the APP-CT positioning.

### FAD-linked PS1 mutations alter APP-CT positioning relative to the membrane

**Figure 3:**
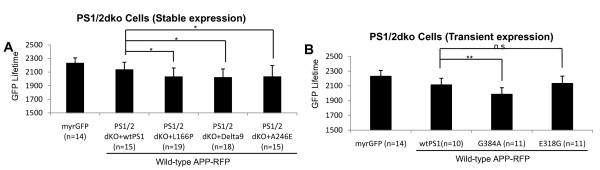
**FAD-linked PS1 mutations alter APP CT positioning **. FLIM analysis of the myrGFP lifetime in PS1/2 dKO cells stably (A) or transiently (B) expressing wild-type or mutant PS1. The cells were co-transfected with myrGFP and wild-type APP-RFP as a FRET pair, and the proximity between myrGFP (membrane) and RFP (APP-CT) was monitored. All FAD-linked PS1 mutations significantly shortened myrGFP lifetime compared to that in the wild-type PS1 expressing cells. The expression of E318G PS1 caused no significant difference in the donor lifetime. Three independent experiments were performed. Data from a representative experiment is shown (n: number of cells; mean ± SD;*p < 0.05; **p < 0.01; ANOVA).

We have previously found that FAD-linked PS1 mutants modify PS1 conformation, and as a result alter an alignment of PS1/γ-secretase with the APP substrate [27]. To test whether this may lead to a change in the positioning of APP-CT relative to the membrane, we monitored proximity between the membrane and APP-CT in PS1/2 dKO cell lines, reconstituted by stable expression of either wild-type PS1 or PS1 with FAD-linked mutations (L166P, Delta9, A246E). Indeed, we found that expression of each FAD-linked PS1 mutation caused a change in the APP-CT positioning (shortening of the GFP donor lifetime) (**Figure **3A), similar to that caused by the FAD APP mutations.

To confirm this finding, PS1/2 dKO cells were transiently co-transfected with either wild-type PS1, FAD-linked G384A PS1, or non-pathogenic E318G PS1 polymorphism together with the myrGFP and wild-type APP-RFP. As expected, expression of the G384A mutant PS1 shortened the lifetime of myrGFP, compared to that of the wild-type PS1. On the other hand, expression of the E318G PS1 polymorphism had no effect on the myrGFP lifetime (**Figure **3B), indicating that FAD-linked mutations, but not a benign polymorphism, alter APP-CT orientation relative to the membrane. Thus, these findings demonstrate that altered conformation of the FAD mutant PS1 and resulting "misalignment" with the APP substrate that leads to increased Aβ_42/40 _ratio, also changes positioning of the APP-CT.

## Discussion

The cytoplasmic domain of APP is believed to function in multiple signalling pathways ranging from apoptosis to gene transcription regulation [35]. APP intracellular domain has been shown to interact with various molecules and contribute to axonal transport [36], neurite outgrowth and arborization [37], and signalling events in the cell [38,39]. Thus, alterations in APP-CT structure in pathological settings could ultimately interfere with these events and accelerate neuropathological changes. A potential relationship between the APP-CT conformation and Aβ production has been suggested. For example, structural studies of the APP-CT have demonstrated that although APP-CT does not adopt a stable folded conformation, it has a transient preordered structure, whose conformation can be altered by phosphorylation [40,41]. In addition, a recent structural model predicts that APP-CT might be associated with the membrane, and suggests that APP-CT association with and dissociation from the membrane might regulate interactions of APP with various proteins [31], and could contribute to an altered Aβ_42/40 _ratio. A new γ-secretase activating protein has been recently described that interacts with both PS1 and APP C-terminal fragment (but not with the Notch substrate), affects Aβ production, and may alter the structural relationship between γ-secretase and APP CT [42].

A model in which there is successive release of tri-peptides has been proposed for differential production of the Aβ_40 _and Aβ_42 _species [43,44]. According to this model, Aβ_49 _produced by PS1/γ-secretase dependent ε-cleavage of APP at the membrane-cytosol interface is converted to Aβ_40 _after successive release of tri-peptides, whereas initial ε-cleavage of APP at the Aβ_48 _site is converted to the Aβ_42_. In the present study, we employed the FLIM assay in intact cells to demonstrate that APP-CT positioning relative to the membrane, or a conformational change of the APP/C99 cytoplasmic domain, correlates with changes in the Aβ_42/40 _ratio. We found that Aβ_42/40 _ratio-raising mutations in PS1 or in the APP transmembrane region altered APP positioning within the membrane by bringing the APP C-terminus closer to the membrane. Thus, a conformational change of the APP cytoplasmic domain, which we observed in the current study, may affect the initial APP cleavage at the ε-site by altering APP substrate presentation to PS1/γ-secretase at the membrane-cytosol interface.

Interestingly, we found that PS1/γ-secretase itself has a profound effect on APP-CT positioning relative to the membrane. First, our data indicate that in the absence of PS1/γ-secretase or when APP-PS1/γ-secretase interaction is inhibited by HP treatment, FRET between APP-CT-RFP and myrGFP-membrane is absent, suggesting that the distal part of the APP-CT is located relatively far away from the membrane. Surprisingly, it appears as if interaction of APP with the PS1/γ-secretase affects the orientation of APP-CT by bringing it into close proximity to the membrane (FRET present). Moreover, interactions with FAD mutant PS1/γ-secretase further change the positioning of the APP-CT to obtain an even closer proximity relative to the membrane. Although the precise mechanism of how mutations in PS1 affect APP positioning relative to the membrane is unknown, it is possible that in the process of APP substrate alignment with the topographically altered mutant γ-secretase active site, changes in the APP-CT membrane proximity occur. This is in agreement with a cross-linking experiment demonstrating that aggressive FAD-linked PS1 mutations cause alterations in topography of the γ-secretase active site [45].

GSMs have been shown to affect both PS1 conformation [13,24,26-28], as well as APP positioning in the membrane (current study). There remains an uncertainty over the primary target of GSMs, with some studies showing that GSMs target γ-secretase, either PS1 itself or other components, such as Pen2, [21,22,42], whereas others propose that GSMs directly bind to the APP substrate [18-20]. In our current study, we did not observe any effect of GSMs on APP-CT positioning in the absence of PS1 and PS2. However, we could not exclude the possibility that GSMs binding may have a subtle effect within the range of non-FRETing distance (>10 nm) from the membrane in PS1/2 dKO cells, thus rendering the APP-CT positioning change undetectable by our current method. It is also possible that some GSMs could still bind to APP CTF in the absence of presenilins but it requires the complex formation for the conformational shift to occur. We have recently reported that the modulatory effect of GSMs is implemented through the "allosteric site" located within the γ-secretase complex itself, although substrate docking to γ-secretase is needed to allow GSM access to this site [24]. Thus, the most likely scenario is that these GSMs primarily target PS1/γ-secretase or the PS1/APP interface, and the change in APP positioning within the membrane is a secondary response to the change of PS1 conformation.

## Conclusions

**Figure 4:**
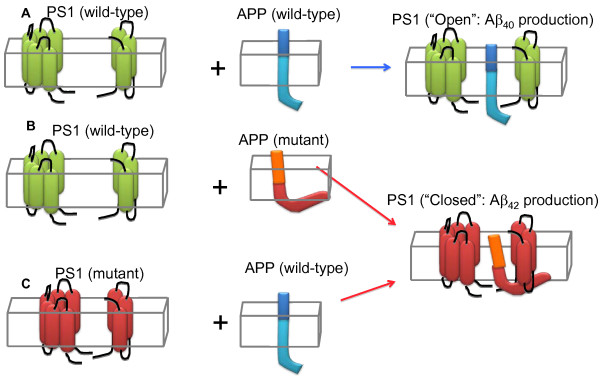
**A scheme of reciprocal interaction between APP-CT positioning relative to the membrane and PS1 conformation **. Wild type PS1/γ-secretase can exist in an "open" and "close" conformational states that correlate with production of Aβ40 and Aβ42, respectively (Lleo, 2004, Berezovska 2005), with the "open" conformation being a predominant state (A). The C-terminus of APP bearing FAD mutation(s) associated with the increased Aβ_42/40 _ratio (orange/red) positions closer to the membrane (B), compared to that in the wild type APP (A). When incorporated into wild type PS1/γ-secretase, it can induce conformational change of PS1 by bringing PS1 NT and CT closer together [25]. On the other hand, when wild type APP is incorporated into FAD-mutant PS1/γ-secretase that predominantly exist in a "closed" conformation, the position of APP C-terminus changes and comes closer to the membrane (C).

In summary, our data demonstrate that interaction of the APP substrate with PS1/γ-secretase changes APP CT positioning relative to the membrane. Moreover, both APP-targeting and PS1-targeting manipulations that change the Aβ_42/40 _ratio can affect APP orientation relative to the membrane as well as PS1 conformation, indicating that APP-CT positioning and PS1 conformation are tightly interconnected, and are in a reciprocal relationship (**Figure **4). Thus, exploring factors affecting PS1 as well as APP conformation would render more insights into the AD pathogenesis, and may provide new information about potential therapeutic targets.

## Methods

### Cell Lines and Pharmacological treatments

PS1/PS2 double knockout (PS1/2 dKO) mouse embryonic fibroblasts (MEFs), and PS1/2 dKO cells reconstituted by stable expression of wild-type or FAD-linked PS1 (L166P, Delta9 and A246E) were a generous gift from Dr. Bart Destrooper [46]. APP/APLP2 dko MEFs were generous a gift from Dr. Koo. Chinese Hamster Ovary (CHO) cells were obtained from ATCC. Cells were cultured with Opti-MEM (Invitrogen) supplemented with 5% fetal bovine serum. The cells plated into four-chamber slides were transfected with various constructs, and were subjected to microscopy (FRET, FLIM) analyses. To evaluate the effect of GSMs on APP, the cells were treated for 24 hours with either 100 μM fenofibrate or 400 μM ibuprofen. To inhibit the interaction between APP and PS1/γ-secretase, cells were treated for 24 hours with 100 nM helical peptide (a gift from Dr. M. Wolfe, BWH, Boston, MA), which was designed to mimic a portion of the APP transmembrane domain and competes with APP for binding to PS1/γ-secretase [34]. Control cells were treated with a vehicle (either DMSO or ethanol).

### Constructs

Human APP 695 isoform was tagged with either green or red fluorescent protein at its C-terminus to generate APP-GFP and APP-mRFP constructs, respectively. APP mutations (V717I, V717K, I716F) were inserted using Quick Change Site-Directed mutagenesis kit (Stratagene), according to the manufacturer's instructions. GFP with myristoylation signal at the N-terminus was described previously [33]. EGFP-C3 empty vector (Clontech, Madison, WI) was used as a control for expression of EGFP without a membrane targeting signal. Wild-type PS1, as well as E318G and G384A mutant PS1 constructs were described previously [27].

### Fluorescence Lifetime Imaging Microscopy (FLIM)

FLIM was used as an approach to monitor proximity between the myrGFP labeled membrane and APP-CT-RFP. Briefly, cells expressing myrGFP only were used as a negative control to determine the baseline myrGFP lifetime. The degree of GFP donor lifetime shortening due to presence of FRET was used as an indicator of the proximity between the GFP donor and RFP acceptor fluorophores in myrGFP and APP-RFP cotransfected cells. The FLIM software uses the Levenberg-Marquardt algorithm to fit the raw data from each pixel to two-exponential fluorescence decay curves to record presence of a shorter than baseline GFP lifetime (for details please see [24,27].

Alternatively, the plasma membrane was labelled with CM-DiI (Molecular Probes) as an acceptor fluorophore in the FLIM assay, and GFP-donor was fused to the APP CT. In this case, cells expressing APP-CT-GFP but not labelled with the CM-DiI served as a negative control. To label the membrane, cells were incubated with 1 μg/ml of CM-DiI dissolved in PBS for 15 minutes at 4°C and fixed with 4% paraformaldehyde, prior to the FLIM analysis. Data analysis was performed using SPC Image (Becker&Hickl, Berlin, Germany), in which donor fluorophore lifetimes are determined by fitting the data to one (negative control) or two (experimental conditions) exponential decay curves. In two component analysis, GFP lifetime (negative control with no acceptor fluorophore) is monitored first, and its value is "fixed" as t1 lifetime. The second, shorter lifetime representing FRET is calculated by the system as t2 value. This t2 value was used for comparisons between different experimental conditions. Thus, "non-FRETing" component (t1 lifetime representing APP molecules that do not interact with PS1, and thus position in the membrane in such a way that does not support FRET) is excluded from the lifetime comparisons.

### ELISA

To measure the effect of APP mutations on Aβ production, wild-type APP-RFP, or APP-RFP, with V717I, V717K or I716F mutations were transfected into CHO cells cultured in 35 mm dishes. 6 hours after the transfection, culture medium was exchanged with 1 ml of fresh OPTI-MEM with 1% FBS, and cells were grown for an additional 24 hours. The conditioned medium was subjected to ELISA analysis using human β-amyloid (1-40 and 1-42) ELISA kit (WAKO, JAPAN), according to the manufacturer's instruction.

### Statistical analysis

StatView for Windows, Version 5.0.1 (SAS Institute, Inc) was employed to perform statistical analysis using Fisher's PSLD analysis of variance (ANOVA). Samples were considered significantly different at p < 0.05.

## Competing interests

The authors declare that they have no competing interests.

## Authors' contributions

KU and PJ carried out the FLIM study. KU analyzed the data and drafted the manuscript. KCF carried out the ELISA, NNG performed western blotting. OB conceived the study, and participated in its design, discussion of the data, and helped to write the manuscript. All authors read and approved the final manuscript.

## Supplementary Material

Additional file 1 **Inhibition of APP-PS1/g-secretase interaction, and not the level of expression, alters APP-CT positioning to the membrane**. (Figure S1)- Level of expression of the wild type (wt), and V717I, I716F and V717K APP mutants in APP/APLP2 dko cells. Level of expression of the wild type APP-RFP (Figure S2) or PS1 (Figure S3) does not alter APP C-terminus positioning relative to membrane, as detected by the FLIM assay (graph in B and C). (Figure S4)- FLIM analysis of APP/APLP2 dKO cells co-transfected with myrGFP and wild-type APP-RFP, and treated with either DMSO or docking site γ-secretase inhibitor, HP. The lifetime of donor myrGFP was significantly longer in cells treated with the HP, compared to that in cells treated with the DMSO (mean ± SD; * p < 0.05, ANOVA). Results from three independent experiments are shown. (n: number of cells examined.) Click here for file
