# Supplementary material for: Reciprocal relationship between APP positioning relative to the membrane and PS1 conformation
Source: Mol Neurodegener. 2011 Feb 10;6:15. doi: 10.1186/1750-1326-6-15 (PMC3046905; doi:10.1186/1750-1326-6-15)
Supplement: Additional file 1 — Inhibition of APP-PS1/g-secretase interaction, and not the level of expression, alters APP-CT positioning to the membrane. (Figure S1)- Level of expression of the wild type (wt), and V717I, I716F and V717K APP mutants in APP/APLP2 dko cells. Level of expression of the wild type APP-RFP (Figure S2) or PS1 (Figure S3) does not alter APP C-terminus positioning relative to membrane, as detected by the FLIM assay (graph in B and C). (Figure S4)- FLIM analysis of APP/APLP2 dKO cells co-transfected with myrGFP and wild-type APP-RFP, and treated with either DMSO or docking site γ-secretase inhibitor, HP. The lifetime of donor myrGFP was significantly longer in cells treated with the HP, compared to that in cells treated with the DMSO (mean ± SD; * p < 0.05, ANOVA). Results from three independent experiments are shown. (n: number of cells examined.) [file 1750-1326-6-15-S1.PPT]

## Slide 1
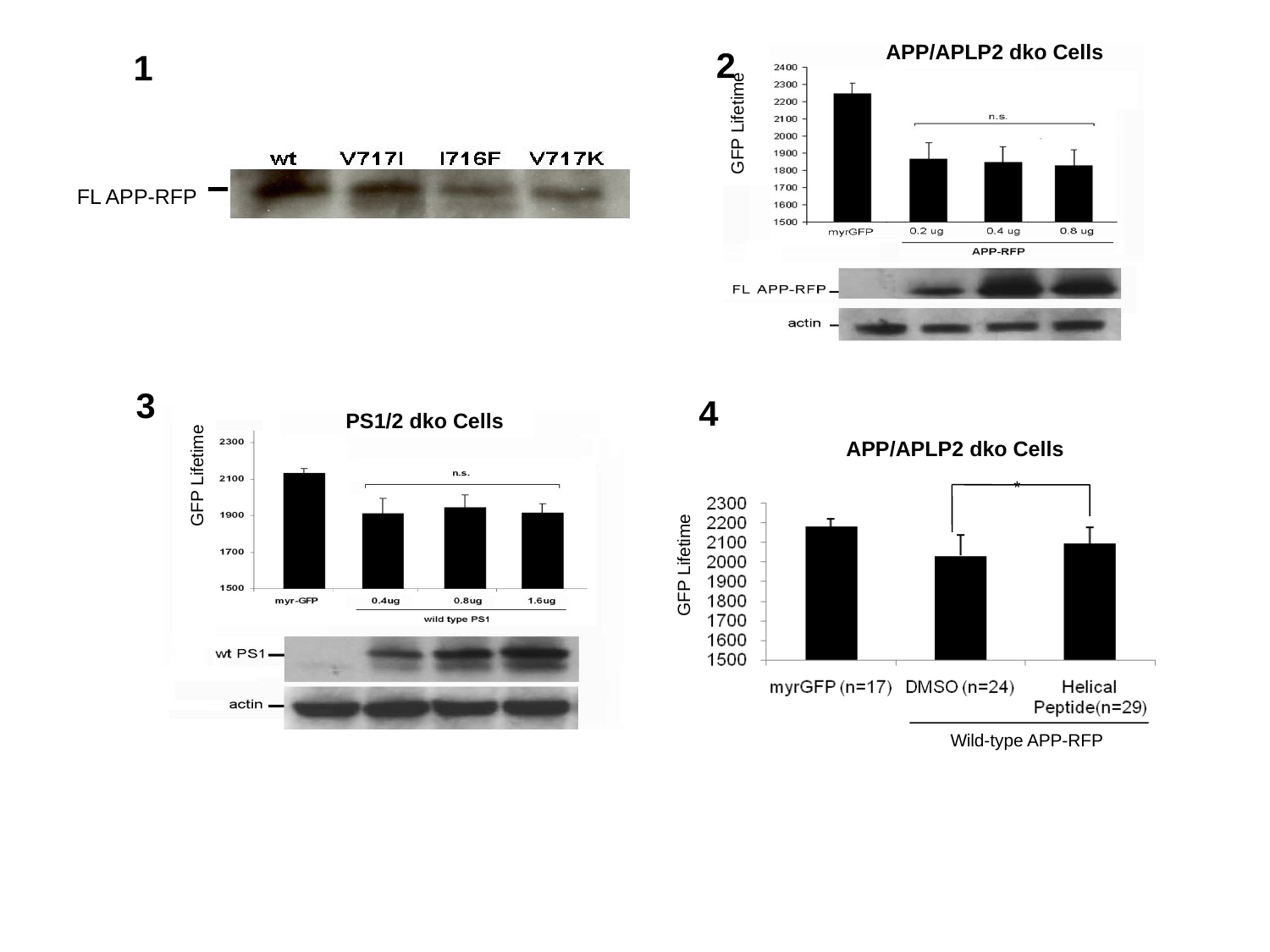

APP/APLP2 dko Cells
2
GFP Lifetime
1
FL APP-RFP
3
GFP Lifetime
PS1/2 dko Cells
4
APP/APLP2 dko Cells
*
GFP Lifetime
Wild-type APP-RFP
